# Characterizing patient-reported outcomes in veterans with cirrhosis

**DOI:** 10.1371/journal.pone.0238712

**Published:** 2020-09-11

**Authors:** Shari S. Rogal, Vera Yakovchenko, Rachel Gonzalez, Angela Park, Carolyn Lamorte, Sandra P. Gibson, Maggie Chartier, David Ross, Emily Comstock, Jasmohan S. Bajaj, Timothy R. Morgan

**Affiliations:** 1 Center for Health Equity Research and Promotion, VA Pittsburgh Healthcare System, Pittsburgh, Pennsylvania, United States of America; 2 Departments of Medicine and Surgery, University of Pittsburgh, Pittsburgh, Pennsylvania, United States of America; 3 Center for Healthcare Organization and Implementation Research, Edith Norse Rogers Memorial VA Hospital, Bedford, Massachusetts, United States of America; 4 Gastroenterology Section, VA Long Beach Healthcare System, Long Beach, California, United States of America; 5 Department of Veterans Affairs, Office of Healthcare Transformation, Washington, DC, United States of America; 6 Veterans Health Administration, HIV, Hepatitis, and Related Conditions Programs, Office of Specialty Care Services, Washington, DC, United States of America; 7 Department of Infectious Diseases, Baltimore VA Medical Center, Baltimore, Maryland, United States of America; 8 Division of Gastroenterology, Hepatology, and Nutrition, Virginia Commonwealth University, Richmond, Virginia, United States of America; 9 Division of Gastroenterology, Hunter Holmes McGuire VA Medical Center, Richmond, Virginia, United States of America; East Carolina University Brody School of Medicine, UNITED STATES

## Abstract

**Background and aims:**

The Veterans Health Administration (VA) cares for over 80,000 Veterans with cirrhosis annually. Given the importance of understanding patient reported outcomes in this complex population, we aimed to assess the associations between attitudes towards care, disease knowledge, and health related quality of life (HRQoL) in a national sample.

**Methods:**

In this cross-sectional study, we mailed paper surveys to a random sample of Veterans with cirrhosis, oversampling those with decompensated disease. Surveys included the Veterans RAND 12-Item Health Survey (measuring HRQoL) and questions about demographics, characteristics of care, satisfaction with care (“attitudes towards care”), and symptoms of cirrhosis. Those who reported being “unsure” about whether they had decompensation events were defined as “unsure about cirrhosis symptoms” (“disease knowledge”). We used multivariable regression models to assess the factors associated with HRQoL.

**Results:**

Of 1374 surveys, 551 (40%) completed surveys were included for analysis. Most Veterans (63%) were “satisfied” or “very satisfied” with VA liver care. Patients often self-reported being unsure about whether they had experienced hepatic decompensation events (34%). Overall average physical (PCS) and mental (MCS) component scores of HRQoL were 30±11 and 41±12. In multivariable regression models, hepatic decompensation (PCS:β = -3.8, MCS:β = -2.2), medical comorbidities (β = −-2.0, β = -1.7), and being unsure about cirrhosis symptoms (β = -1.9, β = -3.3) were associated with worse HRQoL, while age (β = 0.1, β = 0.2) and satisfaction with care (β = 0.6; β = 1.6) were associated with significantly better HRQoL.

**Conclusions:**

Hepatic decompensation, lower satisfaction with care, and being unsure about cirrhosis symptoms were associated with reduced QOL scores in this national cohort.

## Introduction

Quality of life and quality of care have been increasingly linked to health outcomes. Health related quality of life (HRQoL) is broadly defined as the way in which medical illness impacts physical, emotional, and social function [1, 2]. While QOL has been assessed in cirrhosis using a variety of cirrhosis-specific and generic QOL instruments, the value of understanding other key Patient Reported Outcomes (PROs), such as satisfaction with care, has only more recently been recognized in the field of hepatology [3]. PRO is an umbrella term that includes HRQoL, but also patient satisfaction, symptoms of disease, functional status, and treatment compliance [3, 4].

The American Association for the Study of Liver Disease (AASLD) recently recognized the importance of measuring PROs in patients with cirrhosis [3]. AASLD now recommends assessing PROs in patients with cirrhosis across seven domains: physical symptoms, physical function, mental health, general function, cognition, social life, and satisfaction with care. PROs have the potential to reveal important aspects of living with cirrhosis that are often not the primary focus of research or are not accurately perceived by clinicians [2]. Specifically, HRQoL, health distress, sleep disturbance and perceived stigma have been associated with poor health outcomes for patients with cirrhosis [5]. PROs thus add to our understanding of the otherwise unmeasured aspects of disease burden in cirrhosis and their impact on clinical and health services outcomes.

To continuously improve liver care in the Department of Veterans Affairs (VA), VA’s HIV, Hepatitis and Related Conditions Program Office (HHRC), in collaboration with the Office of Healthcare Transformation, developed the Hepatic Innovation Team (HIT) Collaborative [6, 7]. This learning collaborative consists of regional teams of providers who are conducting quality improvement projects to improve liver care in VA. A key component of these efforts is assessing and addressing PROs. To further these efforts, we developed and deployed a national survey for Veterans with cirrhosis. We aimed to 1) assess PROs in a national sample of Veterans with cirrhosis, including satisfaction with care, symptoms, awareness of cirrhosis symptoms, and HRQoL and 2) understand the factors associated with HRQoL in this cohort. We hypothesized that awareness of cirrhosis symptoms, lower satisfaction with care, and more severe disease would be associated with reduced HRQoL.

## Methods

### Sampling

The VA Pittsburgh Healthcare System IRB reviewed this project and considered this work to quality improvement. As such, the need for written informed consent was waived. However, the survey was entirely voluntary. A national VA database was used to identify all Veterans with ICD-9 and -10 codes for cirrhosis or its complications (S1 Appendix) seen at a VA facility in the prior 18 months. Fourteen patients with cirrhosis were randomly selected from each VA site. ICD-9 and ICD-10 codes were used to identify Veterans with hepatic decompensation, with the goal of oversampling this group. In September 2018, surveys were mailed to patients with self-addressed stamped return envelopes. A second round of mailings was sent to Veterans who did not respond to the initial mailing between February and April 2019. Respondents were excluded from further analysis if they indicated that they had undergone transplant, if they returned the survey saying that it did not apply to them, or if they died in the interim.

### Survey development and content

The mailing contained a cover letter that explained the purpose and the voluntary nature of the survey. The survey and cover letter were reviewed by a team of health services and hepatology subject matter experts for readability and content.

#### Demographics

Patients were asked to self-report demographics (age, gender, race, and ethnicity). Region of primary VA site was defined using standard VA geographical areas: Northeast, West, Midwest, and South.

#### Awareness/Knowledge

To assess cirrhosis symptom awareness or knowledge, respondents reported on their symptoms of liver disease, including ascites, gastrointestinal bleeding, and encephalopathy, in patient-centered language (e.g., “Have you ever had fluid buildup in your belly, called ascites, that you were told is related to liver disease?”). Response options included yes, no, or “unsure.” When they reported being “unsure” about any of these decompensation events, they were defined as “unsure about cirrhosis symptoms” for the purposes of these analyses. The self-reported decompensation events were reported individually and as a sum of “self-reported decompensation symptoms,” from 0 to 3.

#### Liver health and comorbidities

Patients were asked to report on the degree to which liver disease contributed to their overall disease burden. They self-reported on 12 comorbidities from the Self-Administered Comorbidity Questionnaire (SCQ), which is designed to be completed by patients and is based on the Charlson Comorbidity Score [8]. We created a summary score using 11 comorbidities for further analyses, excluding liver disease from the score (since it was the disease of interest) [9].

#### Attitudes towards and experiences with care

Respondents reported on their level of satisfaction with VA liver disease care on a 6-point Likert Scale. Veterans also reported how they most frequently receive liver care (in-person vs. by phone vs. telehealth vs. home care vs. unsure).

#### Quality of life

Permission was obtained to use the Veterans RAND 12-Item Health Survey (VR-12). This brief, generic HRQoL instrument is derived from the SF-36. The Veterans SF-36 is considered to be the primary measure of HRQoL within VA [10]. VR-12 assesses 8 key physical and mental health domains: general health, perceptions, physical functioning, role limitations due to physical and emotional problems, bodily pain, energy-fatigue, social functioning and mental health [10]. Summary scores of the Physical Component Summary (PCS) and Mental Component Summary (MCS) scores are standardized, with a range of 0–100, with a mean score of 50 and standard deviation of 10 in a healthy Veteran population. Higher scores signify better physical and mental health [11]. The VR-12 was chosen given its history of validation and its wide use throughout the VA and Centers for Medicare & Medicaid Services (CMS) [10–12].

### Statistical analysis

Descriptive statistics were used to summarize the survey results, using means and standard deviations or medians and interquartile ranges (IQR) for continuous variables and frequencies for categorical variables. PCS and MCS totals were tabulated using standard weights and conversions. The associations between PCS/MCS and covariates were assessed using Spearman’s correlation for continuous covariates and Wilcoxon Rank Sum and Kruskal-Wallace tests for categorical variables. Linear regression models were used to first assess univariate associations between each covariate and PCS/MCS, followed by full models including all variables. Finally, backwards elimination was used to create final models of the factors independently associated with PCS and MCS. The primary analyses included decompensation as a binary (yes/no) variable. In secondary analyses, the number of self-reported decompensation symptoms, ranging from 0 to 3, was substituted for this decompensation variable in the multivariable models. For these analyses, satisfaction was recoded as a numerical variable, using a continuous Likert Scale. Analyses were performed using the R statistical computing environment.

## Results

### Population

**Fig 1:**
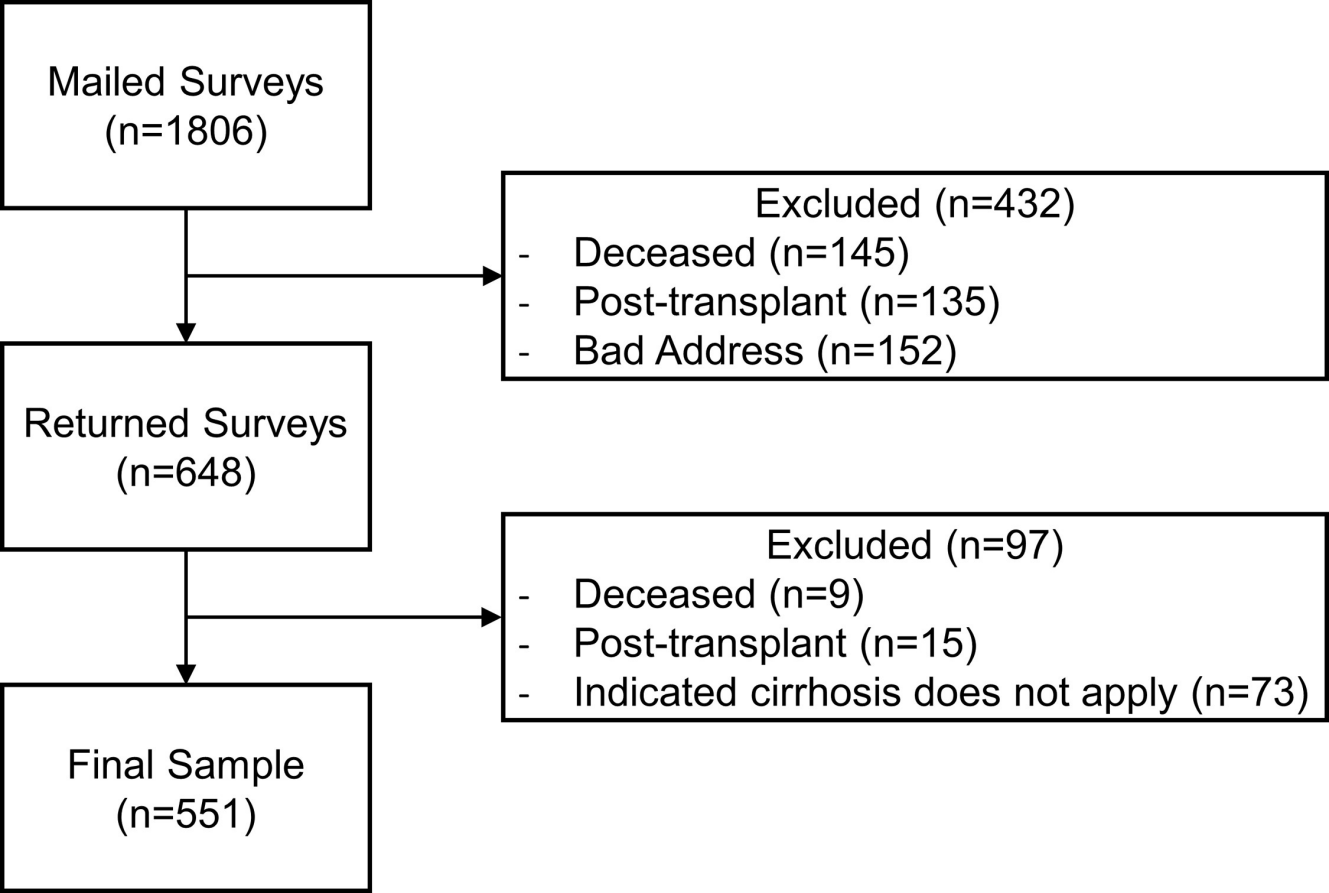
Survey sample.

**Fig 2:**
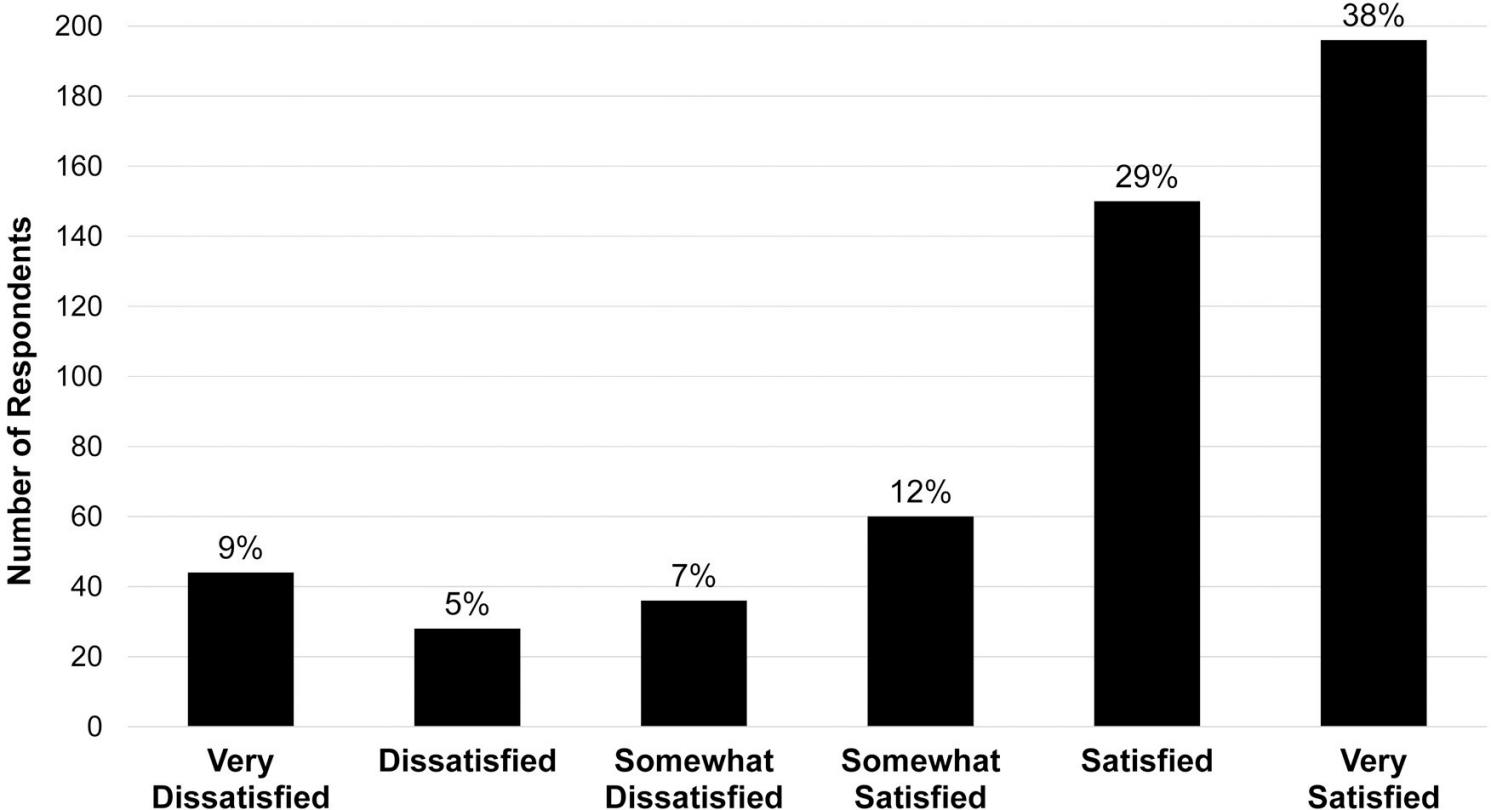
Respondents and locations.

Of 1806 mailed surveys, 432 respondents were subsequently excluded due to interval transplant, death, or non-working address (Fig 1). Of the remaining 1374 surveys, 551 (40%) completed surveys were received from patients with cirrhosis. The respondents were predominantly white (77%) and male (96%) (Table 1). The majority completed the survey independently (89%). Respondents represented from Veterans all VA medical centers and each of four larger geographic “regions” (Fig 2).

**Table 1:** Respondent characteristics (N = 551).

Characteristic	N (%)^a^ or Mean ± SD
Age	67±8
Gender	
Male	528 (96%)
Female	14 (3%)
Other	1 (<1%)
Race	
White or Caucasian	422 (77%)
Black or African American	79 (14%)
Asian or Asian American	4 (<1%)
American Indian or Alaska Native	19 (3%)
Native Hawaiian or Other Pacific Islander	1 (<1%)
Another Race	13 (2%)
Declined to Answer	16 (3%)
Ethnicity	
Hispanic or Latino	43 (8%)
Not Hispanic or Latino	377 (68%)
Declined to Answer	60 (11%)
Comorbidities	
Back pain	317 (58%)
High blood pressure	314 (57%)
Depression	217 (39%)
Diabetes	208 (38%)
Osteoarthritis, degenerative arthritis	145 (26%)
Heart Disease	131 (24%)
Rheumatoid arthritis	98 (18%)
Kidney disease	90 (16%)
Lung Disease	84 (15%)
Anemia, other blood disease	79 (14%)
Cancer	77 (14%)
Ulcer or stomach disease	72 (13%)
Region	
Midwest	129 (23%)
Northeast	85 (15%)
South	191 (35%)
West	146 (26%)

Abbreviations: SD, standard deviation.

^a^Column %s may not sum to 100% due to missing data.

### Liver health and comorbidities

Respondents had a median of 3 (IQR 2–5) non-liver medical problems, most commonly including back pain, hypertension, and depression (Table 1). Most respondents reported that their health was “fair” or “good” and that liver disease contributed at least “somewhat” to their overall health (Table 2).

**Table 2:** Respondent liver disease and liver care characteristics.

Question	Response Frequency N, (%)*
Number of self-reported decompensation symptoms	
0	265 (48%)
1	137 (25%)
2	101 (18%)
3	48 (9%)
Self-reported encephalopathy (n = 542)	
Yes	155 (28%)
No	208 (56%)
Unsure	78 (14%)
Self-reported bleeding	
Yes	111 (20%)
No	369 (68%)
Unsure	64 (12%)
Self-reported ascites	
Yes	217 (39%)
No	226 (41%)
Unsure	99 (18%)
Unsure about any decompensation symptoms	186 (34%)
Overall health	
Excellent	10 (2%)
Very Good	58 (11%)
Good	175 (32%)
Fair	203 (37%)
Poor	96 (18%)
My liver disease causes…	
**all** my health problems	45 (8%)
**most** of my health problems	121 (23%)
**some** of my health problems	138 (26%)
**a few** of my health problems	98 (18%)
**None** of my health problems	129 (24%)
Location of liver care	
Face-to-face	433 (85%)
Phone	3 (<1%)
Telehealth	6 (1%)
Home care	11 (2%)
Unsure	57 (11%)
Satisfaction with liver care	
Very Dissatisfied	44 (9%)
Dissatisfied	28 (5%)
Somewhat Dissatisfied	36 (7%)
Somewhat Satisfied	60 (12%)
Satisfied	150 (29%)
Very Satisfied	196 (38%)

*Column %s may not sum to 100% due to missing values.

### Awareness of cirrhosis symptoms

Approximately half of the patients had ICD codes for decompensation. However, patients often self-reported being “unsure” about whether they had experienced hepatic decompensation events (34%) (Table 2). Being unsure about cirrhosis symptoms occurred across all patients, unrelated to whether they had had codes for decompensation (33% among those who had decompensation vs. 34% among those who had not had decompensation).

### Attitudes towards and experiences with care

Patients reported high overall satisfaction with liver disease care (Table 2). The most frequent location of care was face-to-face (85%).

### Quality of life

The average quality of life for respondents was a PCS of 30±11 and MCS of 41±12, with higher scores on the VR-12 signifying better QOL. Both components of HRQoL were significantly lower for patients with decompensated vs. compensated disease (PCS = 26 vs. 31, p<0.001 and MCS = 40 vs. 42, p = 0.02), defined by ICD codes for decompensation (Table 3). Being unsure about decompensation was associated with even larger decreases in PCS (25 vs. 30, p<0.001) and MCS (37 vs. 43, p<0.001) than actual decompensation.

**Table 3:** Associations between respondent characteristics and QoL.

Characteristic	PCS	MCS
Age	**0.15**	**0.19**
Gender		
Male	29 (21, 30)	41 (32, 52)
Female	28 (21, 30)	43 (29, 51)
Race		
White	28 (21, 37)	**41 (32, 52)**
Black	29 (22, 38)	**40 (33, 41)**
Other	23 (18, 40)	**33 (28, 41)**
Ethnicity		
Hispanic or Latinx	29 (23, 41)	40 (31, 48)
Not Hispanic or Latinx	28 (21, 37)	41 (33, 53)
Comorbidity Score	**-0.40**	**-0.33**
Region		
Midwest	29 (21, 37)	39 (30, 52)
Northeast	28 (22, 39)	46 (36, 54)
South	28 (21, 36)	41 (31, 52)
West	30 (21, 36)	41 (33, 51)
Decompensation		
No	**31 (23, 40)**	**42 (33, 54)**
Yes	**26 (20, 35)**	**40 (31, 50)**
Unsure about Decompensation		
No	**30 (22, 39)**	**43 (33, 54)**
Yes	**25 (19, 35)**	**37 (29, 47)**
Number of Self-reported Decompensation Symptoms		
0	**32 (24, 42)**	**44 (33, 54)**
1	**28 (21, 36)**	**42 (33, 51)**
2	**23 (18, 32)**	**39 (30, 48)**
3	**23 (19, 29)**	**32 (26, 38)**
Satisfaction with care	**0.20**	**0.30**

Correlation is reported for continuous variables (Spearman’s rank correlation reported); median (IQR) PCS and MCS by category is reported for categorical variables and statistical comparisons are made using Wilcoxon Rank Sum or Kruskal-Wallace tests. Statistically significant associations are reported in bold.

Self-reporting decompensation symptoms and reporting being unsure about decompensation were significantly associated with reduced PCS and MCS, regardless of type of decompensation (Table 4). The median difference in scores between those who reported “yes” vs. “no” to decompensation ranged from -6 to -11 for MCS and -6 to -10 for PCS. The median difference in scores between respondents who were “unsure” and those who reported “no” decompensation ranged from -7 to -12 for MCS and -6 to -9 for PCS.

**Table 4:** Self-reported decompensation symptoms and QoL.

		Survey Responses and median (IQR) PCS and MCS for each group			
Question		Yes	No	Unsure	p-values^a^
Have you ever had confusion related to liver disease requiring you to take medications such as lactulose or rifaximin?	N	155	308	78	
	PCS	23 (18, 30)	33 (24, 41)	26 (22, 34)	<0.001
	MCS	36 (28,45)	47 (35,55)	35 (29,45)	<0.001
Have you ever had vomiting of blood or black tarry stools that you were told was due to bleeding from your liver disease?	N	111	369	64	
	PCS	25 (19,34)	31 (21,39)	22 (18,29)	<0.001
	MCS	37 (29,47)	43 (33,54)	36 (28,46)	<0.001
Have you ever had fluid buildup in your belly, called ascites, that you were told is related to liver disease?	N(%)	217	226	99	
	PCS	26 (19,34)	33 (24, 42)	27 (20, 37)	<0.001
	MCS	38 (30,49)	46 (36,55)	36 (30, 47)	<0.001

Abbreviations: IQR, interquartile range; PCS, Physical Component Summary; MCS, Mental Component Summary.

^a^p-values are for comparison of PCS or MCS across responses within each decompensation category by Kruskal-Wallis tests.

In multivariable regression models (Table 5), hepatic decompensation (PCS:β = -3.8, MCS:β = -2.2), medical comorbidities (β = −-2.1, β = -1.7), and disease knowledge, or being unsure about cirrhosis symptoms (β = -1.9, β = -3.3) were significantly associated with worse HRQoL, while age (β = 0.1, β = 0.2) and greater satisfaction with care (β = 0.6; β = 1.6) were significantly associated with significantly improved QOL. There were no significant associations between PCS and demographic characteristics. However, region and race were associated with MCS in the final model (β = 4.7 for Northeast vs. Midwest and β = -4.6 for other race vs. white race).

**Table 5:** Regression models of factors associated with HRQoL in cirrhosis.

Covariates	Full PCS Model	Reduced PCS	Full MCS Model	Reduced MCS
	β(SE)	β(SE)	β(SE)	β(SE)
Age	**0.11 (0.05)**	**0.13 (0.05)**	**0.21 (0.06)**	**0.21 (0.06)**
Male Gender	2.21 (2.84)		-1.06 (3.08)	
Race (vs. white)				
Black	1.92 (1.37)		2.22 (1.49)	2.24 (1.48)
Other	-0.43 (1.89)		**-4.76 (2.04)**	**-4.60 (2.01)**
Hispanic/Latinx Ethnicity	-0.07 (0.87)		-0.35 (0.94)	
Region (vs. Midwest)				
Northeast	1.94 (1.48)		**4.37 (1.61)**	**4.36 (1.61)**
South	0.57 (1.20)		0.97 (1.31)	0.98 (1.30)
West	1.15 (1.28)		2.02 (1.40)	2.01 (1.39)
Comorbidities	**-2.07 (0.23)**	**-2.04 (0.23)**	**-1.72 (0.25)**	**-1.71 (0.25)**
Decompensation yes/no	**-3.66 (0.91)**	**-3.81 (0.89)**	**-2.20 (0.98)**	**-2.22 (0.97)**
Unsure of Decompensation	-1.84 (0.96)	**-1.87 (0.95)**	**-3.28 (1.05)**	**-3.25 (1.04)**
Satisfaction	0.53 (0.29)	**0.57 (0.28)**	**1.55 (0.31)**	**1.55 (0.31)**

Abbreviations: β, regression coefficient; SE, standard error; PCS, Physical Component Summary; MCS, Mental Component Summary Statistically significant relationships are denoted in bold.

In secondary analyses, the number of self-reported decompensation symptoms was substituted for decompensation (yes/no) in the multivariable models. Each additional reported symptom was associated with a reduction of 2.75 points in PCS (p<0.001) and 2.08 points in MCS (p<0.001) in these models, controlling for covariates.

## Discussion

Through this national survey of Veterans of cirrhosis, we found that the strongest predictors of worse HRQoL were younger age, comorbidities, hepatic decompensation, lower satisfaction with care, and being unsure about cirrhosis symptoms. These novel connections between HRQoL and disease knowledge (being unsure about cirrhosis symptoms) and care satisfaction may provide new targets for intervention.

Using a generic HRQoL instrument allows us to compare Veterans with cirrhosis to other disease populations. Persons with no chronic conditions have average physical and mental component scores of 50 and 56, and healthcare-seeking patients have average scores of PCS = 40 and MCS = 50 [12]. Thus, Veterans with cirrhosis had reduced QOL (PCS = 30 and MCS = 41). These findings are consistent with estimates that patients with cirrhosis have score changes of -6 in PCS and -4 in MCS [13]. This cohort had mental component scores as low as patients with depression and physical component scores equivalent to patients with diabetes [12].

While the high burden of physical symptoms in a cohort of patients with cirrhosis was somewhat expected, the high burden of mental health symptoms reinforces how commonplace depression and anxiety symptoms are in patients with cirrhosis [14–16]. In this cross-sectional group of Veterans with cirrhosis, we found that 39% had self-identified depression. Previous studies have identified depression or depressive symptoms in 18–57% of patients with cirrhosis [14, 17–19]. Both depression and anxiety have been associated with HRQoL across disease states (e.g., emphysema and congestive heart failure) [20]. However, while mental health symptoms are clearly associated with the mental component of HRQoL, their association with physical component scores is more nuanced. For example, among 60 patients with cirrhosis, alexithymia, depression, and state- and trait-anxiety were all significantly associated with the mental component of HRQoL, but only trait-anxiety was significantly associated with the physical component [18]. There are several potential mechanisms that may underlie the connection between mental health symptoms and physical component scores. One direct, causal path may be mediated by the association between depression and anxiety and a pro-inflammatory or immunosuppressed state, which may negatively impact other health conditions, such as cirrhosis [19, 21, 22]. Alternatively, depression and anxiety can be associated with negative cognitions that can influence survey responses. These findings again illustrate the importance of screening for and treating mental health symptoms in this population.

Assessing PROs other than HRQoL are important for providing patient-centered care. Satisfaction with health care has recently been described by several prominent guidelines as a critical PRO to measure in patients with cirrhosis [3, 4]. We found that most Veterans were satisfied or highly satisfied with their cirrhosis care and that satisfaction and HRQoL scores are significantly associated. While it is difficult to infer directionality in a cross-sectional study, other longitudinal studies have found an association between satisfaction with care and subsequent HRQoL [23–25]. Patients who are less satisfied with their healthcare care may be less satisfied because their care does not address their physical and mental health symptoms, and this dissatisfaction with suboptimal care then is reflected in lower QOL scores. Conversely, mental health symptoms could theoretically lead to negative cognitive biases that reflect in negative ratings of care. However, regardless of directionality of these associations, addressing patient-reported symptoms not only can improve patients’ satisfaction with care but also can likely positively impact HRQoL.

One unexpected finding was that many patients reported that they were “unsure about cirrhosis symptoms,” including whether they had variceal bleeding, ascites, or encephalopathy symptoms. Being unsure about these decompensation events occurred equally among patients who had and had not had actual decompensation. Low health literacy and disease knowledge among patients with cirrhosis has been previously reported [26–29] and may reflect gaps in health education or literacy. Clinicians often provide inadequate education to patients [27, 30] and overestimate their communication skills around providing health education [31]. This is problematic because health literacy is considered to be “the greatest individual factor affecting a person’s health status” [26, 32]. For patients with cirrhosis, being uncertain about their disease severity or prior complications could have catastrophic consequences, such as not seeking timely care for a variceal bleed.

There are several approaches that may improve HRQoL in patients with cirrhosis. Uncertainty about symptoms of cirrhosis was associated with HRQoL, beyond the decompensation itself. Addressing patients’ health literacy and knowledge around cirrhosis may be one target for addressing uncertainty about decompensation and improving HRQoL. Education interventions (e.g., videos and booklets) [28] can improve knowledge [29, 33] and clinical outcomes [34–36]. Tailored disease self-management programs also show promise in improving care and HRQoL for patients with cirrhosis [36, 37]. VA’s newer “Annie” automated texting program can provide education, facilitate disease self-management, and serve as a platform to collect PROs from patients with liver disease [38]. Multidisciplinary or collaborative care approaches to care also may help address HRQoL in cirrhosis, particularly those programs with a focus on mental health or medication safety [35, 39–41]. However, more research is needed to assess how to optimize disease self-management and HRQoL for patients with cirrhosis.

There are several limitations of the current study, some of which suggest potential areas for future inquiry. First, he cross-sectional design of this study precluded causal inferences or assessments of directionality. Similarly, despite including Veterans from across the country, this study was underpowered to assess clinic-level performance or control for clinic-level care in this study given the small number of Veterans from each site. Moreover, while the 40% response rate is similar to the range of 19–46% reported in other large, national, mailed surveys [42–44], it is unclear whether this biased the findings, either via a “healthy participant bias” or, conversely, due to receiving more responses from dissatisfied or symptomatic patients. Another limitation of the study was the inability to assess health-behaviors, such as adherence, which may mediate other associations and could be of interest in future studies. We selected patients from a VA dashboard that identified patients with cirrhosis using validated ICD-9 and ICD-10 codes. While ICD codes are generally imperfect, the specificity of diagnostic codes in this case is improved since providers at each VA facility can review and remove inappropriate patients from the dashboard. Another potential limitation of the study was the use of self-reported comorbidities. However, self-reported depression in this sample was similar to prior prospective studies [14]. Additionally, the study population included predominantly white males and patients who engaged in face-to-face care, so future work should assess other populations of patients with cirrhosis. Given the unique and unexpected finding that patients who were unsure about their cirrhosis symptoms had lower HRQoL, future work should also directly measure disease knowledge and other potential mediators of this association.

In conclusion, we measured PROs in a national sample of Veterans with cirrhosis from across all VA sites using a validated, generic HRQoL instrument supplemented with questions related to satisfaction with care and medical co-morbidities, symptoms, and certainty about liver-related symptoms. We found that patients with cirrhosis have reduced HRQoL, which was related to not only hepatic decompensation, but also to uncertainty about decompensation events and care satisfaction.

## Supporting information

S1 Appendix Cirrhosis ICD codes. (DOCX)

## Abbreviations

HRQoL: health related quality of life
PRO: patient reported outcome
VA: Department of Veterans Affairs
HHRC: HIV, Hepatitis and Related Conditions Programs
HIT: Hepatic Innovation Team
SCQ: Self-Administered Comorbidity Questionnaire
VR-12: Veterans Rand 12-Item Health Survey
PCS: Physical Component Summary
MCS: Mental Component Summary
