# Supplementary material for: Characterizing patient-reported outcomes in veterans with cirrhosis
Source: PLoS One. 2020 Sep 11;15(9):e0238712. doi: 10.1371/journal.pone.0238712 (PMC7485762; doi:10.1371/journal.pone.0238712)
Supplement: S1 Appendix — (DOCX) [file pone.0238712.s001.docx]

**Appendix Table. Data Definitions**

| \| **ICD Version** \| **ICD Code** \| **ICD Description** \| **Decompensation Code** \| \| --- \| --- \| --- \| --- \| \| 9 \| 456.0 \| Esophageal Varices With Bleeding \| D \| \| 9 \| 456.1 \| Esophageal Varices Without Mention Of Bleeding \|  \| \| 9 \| 456.20 \| Esophageal Varices in Diseases Classified Elsewhere, with bleeding \| D \| \| 9 \| 456.21 \| ESOPHAGEAL VARICES IN DISEASES CLASSIFIED ELSEWHERE, WITHOUT MENTION OF BLEEDING \|  \| \| 9 \| 567.23 \| Spontaneous Bacterial Peritonitis (used after FY2006) \| D \| \| 9 \| 571.2 \| Alcoholic Cirrhosis Of Liver \|  \| \| 9 \| 571.5 \| Cirrhosis Of Liver Without Mention Of Alcohol \|  \| \| 9 \| 572.2 \| Hepatic Coma or hepatic encephalopathy \| D \| \| 9 \| 572.3 \| PORTAL HYPERTENSION \|  \| \| 9 \| 572.4 \| HEPATORENAL SYNDROME \| D \| \| 10 \| I85.00 \| Esophageal Varices Without Bleeding \|  \| \| 10 \| I85.01 \| Esophageal Varices With Bleeding \| D \| \| 10 \| I85.10 \| Secondary esophageal varices without bleeding \|  \| \| 10 \| I85.11 \| Secondary esophageal varices with bleeding \| D \| \| 10 \| K65.2 \| Spontaneous Bacterial Peritonitis \| D \| \| 10 \| K70.11 \| ALCOHOLIC HEPATITIS WITH ASCITES \| D \| \| 10 \| K70.30 \| Alcoholic cirrhosis of liver without ascites \|  \| \| 10 \| K70.31 \| Alcoholic cirrhosis of liver with ascites \| D \| \| 10 \| K70.40 \| Alcoholic hepatic failure without coma \|  \| \| 10 \| K70.41 \| Alcoholic hepatic failure with coma \| D \| \| 10 \| K71.51 \| TOXIC LIVER DISEASE WITH CHRONIC ACTIVE HEPATITIS WITH ASCITES \| D \| \| 10 \| K71.7 \| TOXIC LIVER DISEASE WITH FIBROSIS AND CIRRHOSIS OF LIVER \|  \| \| 10 \| K72.10 \| Chronic hepatic failure without coma \|  \| \| 10 \| K72.11 \| Chronic hepatic failure with coma \| D \| \| 10 \| K72.90 \| Hepatic failure, unspecified, without coma \|  \| \| 10 \| K72.91 \| Hepatic failure, unspecified with coma \| D \| \| 10 \| K74.3 \| PRIMARY BILIARY CIRRHOSIS \|  \| \| 10 \| K74.4 \| SECONDARY BILIARY CIRRHOSIS \|  \| \| 10 \| K74.5 \| BILIARY CIRRHOSIS, UNSPECIFIED \|  \| \| 10 \| K74.60 \| Unspecified cirrhosis of liver \|  \| \| 10 \| K74.69 \| Other cirrhosis of liver \|  \| \| 10 \| K76.6 \| Portal hypertension \|  \| \| 10 \| K76.7 \| HEPATORENAL SYNDROME \| D \| \| 10 \| K76.81 \| HEPATOPULMONARY SYNDROME \| D \| |  |
| --- | --- | --- | --- | --- | --- | --- | --- | --- | --- | --- | --- | --- | --- | --- | --- | --- | --- | --- | --- | --- | --- | --- | --- | --- | --- | --- | --- | --- | --- | --- | --- | --- | --- | --- | --- | --- | --- | --- | --- | --- | --- | --- | --- | --- | --- | --- | --- | --- | --- | --- | --- | --- | --- | --- | --- | --- | --- | --- | --- | --- | --- | --- | --- | --- | --- | --- | --- | --- | --- | --- | --- | --- | --- | --- | --- | --- | --- | --- | --- | --- | --- | --- | --- | --- | --- | --- | --- | --- | --- | --- | --- | --- | --- | --- | --- | --- | --- | --- | --- | --- | --- | --- | --- | --- | --- | --- | --- | --- | --- | --- | --- | --- | --- | --- | --- | --- | --- | --- | --- | --- | --- | --- | --- | --- | --- | --- | --- | --- | --- | --- | --- | --- | --- | --- | --- | --- | --- | --- | --- | --- | --- |

To receive a diagnosis of cirrhosis, patients had to have 2 outpatient or one inpatient code for the following. Codes with a D in the right column qualified as “decompensation” codes for the purposes of this project.
